# On blow-up criteria for a coupled chemotaxis fluid model

**DOI:** 10.1186/s13660-017-1304-4

**Published:** 2017-01-31

**Authors:** Hongyan Xie, Caochuan Ma

**Affiliations:** 1 School of Economics, Southwestern University of Finance and Economics, Chengdu, 611130 P.R. China; 2 Department of Mathematics, Tianshui Normal University, Tianshui, 741000 Ganshu P.R. China

**Keywords:** 22E46, 53C35, 57S20, Navier-Stokes, chemotaxis, oxygentaxis, regularity

## Abstract

We consider a coupled chemotaxis fluid model and prove some blow-up criteria of the local strong solution.

## Introduction

We consider the following coupled chemotaxis fluid model [1]:

1.1 $$\begin{aligned}& u_{t}+(u\cdot\nabla) u+\nabla\pi-\Delta u+n\nabla\phi=0, \end{aligned}$$

1.2 $$\begin{aligned}& \operatorname{div}u=0, \end{aligned}$$

1.3 $$\begin{aligned}& n_{t}+(u\cdot\nabla)n-\Delta n=-\nabla\cdot\bigl(n\chi(p)\nabla p \bigr), \end{aligned}$$

1.4 $$\begin{aligned}& p_{t}+(u\cdot\nabla)p=-nf(p), \end{aligned}$$

1.5 $$\begin{aligned}& (u,n,p) (x,0)=(u_{0},n_{0},p_{0}) (x) \quad \mbox{in } \mathbb{R}^{3}. \end{aligned}$$

Here *u* denotes the velocity vector field of the fluid and *π* is the pressure scalar, *p* and *n* denote the concentration of oxygen and bacteria, respectively. ∇*ϕ* is the gravitation force. $f(p)\geq f(0)=0$ and $\chi(p)\geq0$ are two given smooth functions of *p*.

When $\phi=0$, (1.1) and (1.2) are the well-known Navier-Stokes system. Kozono *et al.* [2] and Kozono and Shimada [3] proved the following blow-up criteria:

1.6 $$\begin{aligned}& u\in L^{2}\bigl(0,T;\dot{B}^{0}_{\infty,\infty} \bigr), \end{aligned}$$

1.7 $$\begin{aligned}& u\in L^{\frac{2}{1-\theta}}\bigl(0,T;\dot{B}^{-\theta}_{\infty,\infty} \bigr)\quad \mbox{with }0< \theta< 1, \end{aligned}$$

1.8 $$\begin{aligned}& \omega:=\operatorname{curl}u\in L^{1}\bigl(0,T; \dot{B}^{0}_{\infty,\infty}\bigr). \end{aligned}$$

Here $\dot{B}^{s}_{p,q}$ denotes the homogeneous Besov space. Zhang *et al.* [4] showed the following blow-up criterion in terms of pressure:

1.9 $$ \pi\in L^{\frac{2}{2+r}}\bigl(0,T;\dot{B}^{r}_{\infty,\infty} \bigr)\quad\mbox{with }{-}1\leq r\leq1. $$

When $u=\nabla\phi=0$, (1.3) and (1.4) are the Keller-Segel model which was studied in [5, 6].

Very recently, Chae *et al.*[7] showed the local well-posedness of smooth solutions to problem (1.1)-(1.5) and the following blow-up criterion:

1.10 $$ u\in L^{\frac{2q}{q-3}}\bigl(0,T;L^{q}\bigr)\quad \mbox{and}\quad n\in L^{2}\bigl(0,T;L^{\infty}\bigr)\quad \mbox{with }3< q\leq\infty. $$

The aim of this paper is to refine (1.10) further; we will prove the following.

### Theorem 1.1

*Let the initial data* $(u_{0},n_{0},p_{0})$ *be given in* $H^{l}\times H^{l-1}\times H^{l}$ *for* $l>\frac{5}{2}$ *and* $n_{0}$, $p_{0}\geq0$ *in* $\mathbb{R}^{3}$ *and* $\int_{\mathbb{R}^{3}}n_{0}\,dx<\infty$. *Suppose that* *ϕ* *is a smooth function*. *Let* $(u, n, p)$ *be a local smooth solution on* $[0,\tilde{T})$ *for some* $\tilde{T}\leq\infty$. *If* *u* *satisfies* (1.6) *or* (1.7) *or* (1.8) *or* *π* *satisfies* (1.9) ($r=-1$) *and* *n* *satisfies*

1.11 $$ n\in L^{2}\bigl(0,T;L^{\infty}\bigr) $$

*with* $\tilde{T}\leq T<\infty$, *then the solution* $(u, n ,p)$ *can be extended beyond* $T>0$.

### Corollary 1.1

*If u satisfies* (1.6) *or* (1.7) *or* (1.8) *or* *π* *satisfies* (1.9) *and* ∇*p* *satisfies*

1.12 $$ \nabla p\in L^{\frac{2q}{q-3}}\bigl(0,T;L^{q}\bigr)\quad \textit{with }3< q\leq\infty, $$

*with* $\tilde{T}\leq T<\infty$, *then the solution* $(u, n ,p)$ *can be extended beyond* $T>0$.

### Remark 1.1

By the very same calculations as those in Zhou [8], we can prove the following blow-up criteria:

1.13 $$ \pi\in L^{\frac{2q}{2q-3}}\bigl(0,T;L^{q}\bigr)\quad \mbox{with }3/2< q\leq\infty, $$

or

1.14 $$ \nabla\pi\in L^{\frac{2q}{3q-3}}\bigl(0,T;L^{q}\bigr)\quad \mbox{with }1< q\leq\infty, $$

and *n* satisfies (1.11). We omit the details here.

## Preliminary

Here we recall the definitions and some properties of spaces.

Let $\mathfrak{B}=\{\xi\in\mathbb{R}^{d}, \vert \xi \vert \le \frac{4}{3}\}$ and $\mathfrak{C}=\{\xi\in\mathbb{R}^{d}, \frac{3}{4}\le \vert \xi \vert \le\frac{8}{3}\}$. Choose two nonnegative smooth radial functions *χ*, *φ* supported, respectively, in $\mathfrak{B}$ and $\mathfrak{C}$ such that

$$\begin{gathered} \chi(\xi)+\sum_{j\ge0}\varphi\bigl(2^{-j}\xi \bigr)=1,\quad\xi\in\mathbb{R}^{d}, \\ \sum_{j\in\mathbb{Z}}\varphi\bigl(2^{-j}\xi\bigr)=1, \quad\xi\in\mathbb {R}^{d}\setminus\{0\}. \end{gathered} $$

We denote $\varphi_{j}=\varphi(2^{-j}\xi)$, $h=\mathfrak{F}^{-1}\varphi $ and $\tilde{h}=\mathfrak{F}^{-1}\chi$, where $\mathfrak{F}^{-1}$ stands for the inverse Fourier transform. Then the dyadic blocks $\Delta_{j}$ and $S_{j}$ can be defined as follows:

$$\begin{gathered} \Delta_{j}f=\varphi\bigl(2^{-j}D\bigr)f=2^{jd} \int_{\mathbb{R}^{d}}h\bigl(2^{j}y\bigr)f(x-y)\,dy, \\ S_{j}f=\sum_{k\le j-1}\Delta_{k}f= \chi\bigl(2^{-j}D\bigr)f=2^{jd} \int_{\mathbb {R}^{d}}\tilde{h}\bigl(2^{j}y\bigr)f(x-y)\,dy. \end{gathered} $$

Formally, $\Delta_{j}=S_{j}-S_{j-1}$ is a frequency projection to annulus $\{\xi: C_{1}2^{j}\le \vert \xi \vert \le C_{2}2^{j}\}$, and $S_{j}$ is a frequency projection to the ball $\{\xi: \vert \xi \vert \le C2^{j}\}$. One can easily verify that, with our choice of *φ*,

$$\Delta_{j}\Delta_{k}f=0\quad \mbox{if } \vert j-k\vert \ge2\quad \mbox{and}\quad \Delta_{j}(S_{k-1}f\Delta_{k}f)=0 \quad \mbox{if }\vert j-k\vert \ge5. $$

With the introduction of $\Delta_{j}$ and $S_{j}$, let us recall the definition of the Besov space.

### Definition 2.1

[9, 10]

Let $s\in\mathbb{R}$, $(p,q)\in [1,\infty]^{2}$, the homogeneous space $\dot{B}_{p,q}^{s}$ is defined by

$$\dot{B}_{p,q}^{s}=\bigl\{ f\in\mathfrak{S}'; \Vert f\Vert _{\dot {B}_{p,q}^{s}}< \infty\bigr\} , $$

where

$$ \Vert f\Vert _{\dot{B}_{p,q}^{s}}= \textstyle\begin{cases} (\sum _{j\in\mathbb{Z}}2^{sqj}\Vert \Delta_{j}f \Vert _{L^{p}}^{q})^{\frac{1}{q}},&\mbox{for } 1\le q< \infty, \\ \sup_{j\in\mathbb{Z}}2^{sj}\Vert \Delta_{j}f \Vert _{L^{p}},&\mbox{for } q=\infty, \end{cases} $$

and $\mathfrak{S}'$ denotes the dual space of $\mathfrak{S}=\{f\in \mathcal{S}(\mathbb{R}^{d}); \partial^{\alpha}\hat{f}(0)=0; \forall\alpha \in \mathbb{N}^{d} \mbox{ multi-index}\}$ and can be identified by the quotient space of $\mathcal{S'}/\mathcal{P}$ with the polynomials space $\mathcal{P}$.

### Lemma 2.1

[4]

*Let a measurable function* *π* *satisfy*

$$\pi\in\dot{B}_{\infty,\infty}^{r}\bigl(\mathbb{R}^{3}\bigr) $$

*for some* *r* *with* $-1\leq r\leq1$, *then there exists a decomposition* $\pi:=\pi_{\ell}+\pi_{h}$ *such that*

$$\nabla^{2}\pi_{\ell}\in L^{\infty}\bigl( \mathbb{R}^{3}\bigr)\quad \textit{and}\quad \pi _{h}\in W^{-1,\infty}\bigl(\mathbb{R}^{3}\bigr), $$

*and*

$$\begin{aligned}& \bigl\Vert \nabla^{2}\pi_{\ell}\bigr\Vert _{L^{\infty}}^{\frac{1}{2}}+\Vert \pi_{h}\Vert _{ W^{-1,\infty}}^{2}\leq C\bigl(e+\Vert \pi \Vert _{\dot{B}_{\infty,\infty}^{r}}^{\frac{2}{2+r}}\bigr), \\& \Vert \pi_{\ell} \Vert _{L^{2}}\leq C\Vert \pi \Vert _{L^{2}}, \Vert \nabla\pi_{h}\Vert _{L^{2}}\leq C \Vert \nabla \pi \Vert _{L^{2}}. \end{aligned}$$

## Proof of Theorem 1.1

This section is devoted to the proof of Theorem 1.1. Since local existence results have been proved in [7], we only need to prove *a priori* estimates.

To begin with, it is easy to see that

3.1 $$ n\geq0,\qquad 0\leq p\leq C,\qquad \int_{\mathbb{R}^{3}}n\,dx= \int_{\mathbb {R}^{3}}n_{0}\,dx< \infty. $$

Case 1. Let (1.6) and (1.11) hold true.

Testing (1.1) by *u* and using (1.2), we infer that

$$\begin{aligned} \frac{1}{2}\frac{d}{dt} \int_{\mathbb{R}^{3}}\vert u\vert ^{2}\,dx+ \int_{\mathbb{R}^{3}}\vert \nabla u\vert ^{2}\,dx =& \int _{\mathbb{R}^{3}}n\nabla\phi u\,dx \\ \leq&\Vert n\Vert _{L^{\infty}} \Vert \nabla\phi \Vert _{L^{2}} \Vert u\Vert _{L^{2}}, \end{aligned}$$

which leads to

3.2 $$ \Vert u\Vert _{L^{\infty}(0,T;L^{2})}+\Vert u\Vert _{L^{2}(0,T;H^{1})} \leq C. $$

In the following calculations, we will use the following elegant inequality [11, 12]:

$$\Vert \nabla u\Vert _{L^{4}}^{2}\leq C\Vert u\Vert _{\dot{B}_{\infty,\infty}^{0}}\Vert \Delta u\Vert _{L^{2}}. $$

Testing (1.1) by Δ*u*, using (1.2) and the above inequality, we find that

$$\begin{aligned}& \frac{1}{2}\frac{d}{dt} \int_{\mathbb{R}^{3}}\vert \nabla u\vert ^{2}\,dx+ \int_{\mathbb{R}^{3}}\vert \Delta u\vert ^{2}\,dx \\& \quad = \int_{\mathbb{R}^{3}}(u\cdot\nabla)u\cdot\Delta u \,dx+ \int _{\mathbb{R}^{3}}n\nabla\phi\Delta u\,dx \\& \quad = \sum_{i,j} \int_{\mathbb{R}^{3}}u_{i}\partial_{i}u \,\partial_{j}^{2}u\,dx+ \int _{\mathbb{R}^{3}}n\nabla\phi\Delta u\,dx \\& \quad = -\sum_{i,j} \int_{\mathbb{R}^{3}}\partial_{j}u_{i} \,\partial_{i}u\partial _{j}udx+ \int_{\mathbb{R}^{3}}n\nabla\phi\Delta u\,dx \\& \quad \leq C\Vert \nabla u\Vert _{L^{4}}^{2}\Vert \nabla u\Vert _{L^{2}}+\Vert n\Vert _{L^{\infty}} \Vert \nabla\phi \Vert _{L^{2}}\Vert \Delta u\Vert _{L^{2}} \\& \quad \leq C\Vert u\Vert _{\dot{B}_{\infty,\infty}^{0}}\Vert \Delta u\Vert _{L^{2}}\Vert \nabla u\Vert _{L^{2}}+C\Vert n\Vert _{L^{\infty}} \Vert \Delta u\Vert _{L^{2}} \\& \quad \leq\frac{1}{2}\Vert \Delta u\Vert _{L^{2}}^{2}+C \Vert u\Vert _{\dot{B}_{\infty,\infty}^{0}}^{2}\Vert \nabla u\Vert _{L^{2}}^{2}+C\Vert n\Vert _{L^{\infty}}^{2}, \end{aligned}$$

which gives

3.3 $$ \Vert u\Vert _{L^{\infty}(0,T;H^{1})}+\Vert u\Vert _{L^{2}(0,T;H^{2})} \leq C. $$

By (1.10), this completes the proof of Case 1.

Case 2. Let (1.7) and (1.11) hold true.

Testing (1.1) by $-\Delta u$, using (1.2) and the following inequalities [3, 11]:

3.4 $$\begin{aligned}& \Vert u\cdot\nabla u\Vert _{L^{2}}\leq C\Vert u \Vert _{\dot{B}_{\infty,\infty}^{-\theta}} \Vert u\Vert _{\dot {B}_{2,1}^{1+\theta}}, 0< \theta< 1, \end{aligned}$$

3.5 $$\begin{aligned}& \Vert u\Vert _{\dot{B}_{2,1}^{\theta}}\leq C\Vert u\Vert _{L^{2}}^{1-\theta} \Vert \nabla u\Vert _{L^{2}}^{\theta}, 0< \theta< 1, \end{aligned}$$

we derive

$$\begin{aligned}& \frac{1}{2}\frac{d}{dt} \int_{\mathbb{R}^{3}}\vert \nabla u\vert ^{2}\,dx+ \int_{\mathbb{R}^{3}}\vert \Delta u\vert ^{2}\,dx \\& \quad= \int_{\mathbb{R}^{3}}(u\cdot\nabla)u \cdot\Delta u \,dx+ \int_{\mathbb {R}^{3}}n\nabla\phi\Delta u\,dx \\& \quad\leq \Vert u\cdot\nabla u\Vert _{L^{2}}\Vert \Delta u\Vert _{L^{2}}+\Vert n\Vert _{L^{\infty}} \Vert \nabla \phi \Vert _{L^{2}}\Vert \Delta u\Vert _{L^{2}} \\& \quad\leq C\Vert u\Vert _{\dot{B}_{\infty,\infty}^{-\theta }}\Vert \nabla u\Vert _{L^{2}}^{1-\theta} \Vert \Delta u\Vert _{L^{2}}^{1+\theta}+C \Vert n\Vert _{L^{\infty}} \Vert \Delta u\Vert _{L^{2}} \\& \quad\leq\frac{1}{2}\Vert \Delta u\Vert _{L^{2}}^{2}+C \Vert u\Vert _{\dot{B}_{\infty,\infty}^{\frac{2}{1-\theta}}} \Vert \nabla u\Vert _{L^{2}}^{2}+C \Vert n\Vert _{L^{\infty}}^{2}, \end{aligned}$$

which yields (3.3); this completes the proof of Case 2 again by (1.10).

Case 3. Let (1.8) and (1.11) hold true.

Testing (1.1) by $-\Delta u$, using (1.2), we deduce that

3.6 $$ \begin{aligned}[b] &\frac{1}{2}\frac{d}{dt} \int_{\mathbb{R}^{3}}\vert \nabla u\vert ^{2}\,dx+ \int_{\mathbb{R}^{3}}\vert \Delta u\vert ^{2}\,dx \\ &\quad=-\sum_{i,j} \int_{\mathbb{R}^{3}}\partial_{j}u_{i} \,\partial_{i}u\partial _{j}udx+ \int_{\mathbb{R}^{3}}n\nabla\phi\Delta u\,dx \\ &\quad=:I_{1}+ \int_{\mathbb{R}^{3}}n\nabla\phi\Delta u \,dx. \end{aligned} $$

By the very same calculations as those in [13], we get

3.7 $$ I_{1}\leq\frac{1}{8}\Vert \Delta u\Vert _{L^{2}}^{2}+C\Vert \nabla u\Vert _{L^{2}}^{2}+C \Vert \nabla u\Vert _{\dot {B}_{\infty,\infty}^{0}}\Vert \nabla u\Vert _{L^{2}}^{2}\operatorname{log}\bigl(e+\Vert \nabla u\Vert _{L^{2}}^{2}\bigr). $$

Inserting (3.7) into (3.6) and solving the resulting inequality, we arrive at (3.3). This completes the proof of Case 3.

Case 4. Let (1.9) ($r=-1$) and (1.11) hold true.

Testing (1.1) by $\vert u\vert ^{2}u$ and using (1.2), we observe that

3.8 $$ \begin{aligned}[b] &\frac{1}{4}\frac{d}{dt} \int_{\mathbb{R}^{3}}\vert u\vert ^{4}\,dx+ \int_{\mathbb{R}^{3}}\vert u\vert ^{2}\vert \nabla u\vert ^{2}\,dx+\frac{1}{2} \int_{\mathbb{R}^{3}}\bigl\vert \nabla \vert u\vert ^{2} \bigr\vert ^{2}\,dx \\ &\quad=- \int_{\mathbb{R}^{3}}(u\cdot\nabla)\pi \vert u\vert ^{2}\,dx- \int_{\mathbb{R}^{3}}n\nabla\phi \vert u\vert ^{2} u\,dx \\ &\quad=:I_{2}+I_{3}. \end{aligned} $$

$I_{3}$ can be bounded as follows:

3.9 $$ I_{3}\leq \Vert n\Vert _{L^{\infty}} \Vert \nabla \phi \Vert _{L^{4}}\Vert u\Vert _{L^{4}}^{3}. $$

We bounded $I_{2}$ as follows:

3.10 $$\begin{aligned} I_{2}&= \int_{\mathbb{R}^{3}}\pi u \cdot\nabla \vert u\vert ^{2}\,dx \\ &\leq \Vert \pi \Vert _{L^{4}}\Vert u\Vert _{L^{4}}\bigl\Vert \nabla \vert u\vert ^{2}\bigr\Vert _{L^{2}} \\ &\leq \Vert \pi \Vert _{\dot{B}_{\infty,\infty}^{-1}}^{\frac {1}{2}}\Vert \nabla\pi \Vert _{L^{2}}^{1/2}\Vert u\Vert _{L^{4}}\bigl\Vert \nabla \vert u\vert ^{2}\bigr\Vert _{L^{2}} \\ &\leq \Vert \pi \Vert _{\dot{B}_{\infty,\infty}^{-1}}^{\frac {1}{2}}\bigl(\Vert u\cdot\nabla u\Vert _{L^{2}}+\Vert n\nabla \phi \Vert _{L^{2}} \bigr)^{1/2}\Vert u\Vert _{L^{4}}\bigl\Vert \nabla \vert u \vert ^{2}\bigr\Vert _{L^{2}} \\ &\leq \Vert \pi \Vert _{\dot{B}_{\infty,\infty}^{-1}}^{\frac {1}{2}}\bigl(\bigl\Vert u \vert \nabla u\vert \bigr\Vert _{L^{2}}+\Vert n\Vert _{L^{\infty}}\bigr)^{1/2}\Vert u\Vert _{L^{4}}\bigl\Vert \nabla \vert u\vert ^{2}\bigr\Vert _{L^{2}} \\ &\leq\frac{1}{8}\bigl\Vert \nabla \vert u\vert ^{2}\bigr\Vert _{L^{2}}^{2}+\frac{1}{8}\bigl\Vert u\vert \nabla u\vert \bigr\Vert _{L^{2}}^{2}+C\Vert \pi \Vert _{\dot{B}_{\infty,\infty }^{-1}}^{2}\Vert u\Vert _{L^{4}}^{4}+C \Vert n\Vert _{L^{\infty}}^{2}, \end{aligned}$$

where we have used the elegant inequality [11, 12]

3.11 $$ \Vert \pi \Vert _{L^{4}}^{2}\leq C\Vert \pi \Vert _{\dot {B}_{\infty,\infty}^{-1}}\Vert \nabla\pi \Vert _{L^{2}}, $$

and the pressure estimate

3.12 $$ \Vert \nabla\pi \Vert _{L^{2}}\leq C\bigl(\Vert u\cdot \nabla u\Vert _{L^{2}}+\Vert n\nabla\phi \Vert _{L^{2}}\bigr). $$

Inserting (3.9) and (3.10) into (3.8) and using the Gronwall inequality, we conclude that

3.13 $$ \Vert u\Vert _{{L^{\infty}}(0,T;{L^{4}})}\leq C. $$

By (1.10), this completes the proof of Case 4.

## Proof of Corollary 1.1

Testing (1.3) by $n^{m-1}$ ($m\geq2$), using (1.2) and (3.1) and denoting $w:=n^{\frac{m}{2}}$, we have

$$\begin{aligned}& \frac{1}{m}\frac{d}{dt} \int_{\mathbb{R}^{3}}w^{2}\,dx+\frac{4(m-1)}{m^{2}} \int _{\mathbb{R}^{3}}\vert \nabla w\vert ^{2}\,dx \\& \quad\leq C\biggl\vert \int\chi(p)\nabla p\cdot w\nabla w \,dx\biggr\vert \\& \quad\leq C\Vert \nabla p\Vert _{L^{q}}\Vert w\Vert _{L^{\frac{2q}{q-2}}}\Vert \nabla w\Vert _{L^{2}} \\& \quad\leq C\Vert \nabla p\Vert _{L^{q}}\Vert w\Vert _{L^{2}}^{1-\frac{3}{q}}\Vert \nabla w\Vert _{L^{2}}^{1+\frac{3}{q}} \\& \quad\leq\frac{m-1}{m^{2}}\Vert \nabla w\Vert _{L^{2}}^{2}+C \Vert \nabla p\Vert _{L^{q}}^{\frac{2q}{q-3}}\Vert w\Vert _{L^{2}}^{2}, \end{aligned}$$

which implies

4.1 $$ \Vert n\Vert _{{L^{\infty}}(0,T;{L^{m}})}\leq C\quad\mbox{for }m>2. $$

Here we used the Gagliardo-Nirenberg inequality

4.2 $$ \Vert w\Vert _{L^{\frac{2q}{q-2}}}\leq C\Vert w\Vert _{L^{2}}^{1-\frac{3}{q}}\Vert \nabla w\Vert _{L^{2}}^{\frac {3}{q}} \quad\mbox{with }3< q\leq\infty. $$

Now, since the proofs of other cases are very similar to those in Case 1, Case 2, Case 3 and Case 4, we only prove the following case: Let (1.9) ($-1< r\leq1$) and (1.12) hold true.

We still have (3.8) and (3.9).

Using Lemma 2.1, (3.11), (3.12) and the pressure estimate

4.3 $$ \begin{aligned}[b] \Vert \pi \Vert _{L^{2}}& \leq C\bigl(\Vert u\Vert _{L^{4}}^{2}+\bigl\Vert (- \Delta)^{-\frac{1}{2}}(n\nabla\phi)\bigr\Vert _{L^{2}}\bigr) \\ &\leq C\bigl(\Vert u\Vert _{L^{4}}^{2}+\Vert n\nabla\phi \Vert _{L^{\frac{6}{5}}}\bigr) \\ &\leq C\bigl(\Vert u\Vert _{L^{4}}^{2}+1\bigr), \end{aligned} $$.

we bound $I_{2}$ as follows:

4.4 $$ \begin{aligned}[b] I_{2}&=- \int_{\mathbb{R}^{3}}u\nabla\pi_{\ell} \vert u\vert ^{2}\,dx- \int_{\mathbb{R}^{3}}u\nabla\pi_{h}\vert u\vert ^{2}\,dx \\ &\leq \Vert \nabla\pi_{\ell} \Vert _{L^{4}}\Vert u\Vert _{L^{4}}^{3}+ \int_{\mathbb{R}^{3}}u\pi_{h}\nabla \vert u\vert ^{2}\,dx \\ &\leq \Vert \pi_{\ell} \Vert _{L^{2}}^{\frac{1}{2}}\bigl\Vert \nabla^{2}\pi_{\ell}\bigr\Vert _{L^{\infty}}^{{\frac{1}{2}}} \Vert u\Vert _{L^{4}}^{3}+\Vert u\Vert _{L^{4}} \Vert \pi _{h}\Vert _{L^{4}}\bigl\Vert \nabla \vert u \vert ^{2}\bigr\Vert _{L^{2}} \\ &\leq \Vert \pi_{\ell} \Vert _{L^{2}}^{\frac{1}{2}}\bigl\Vert \nabla^{2}\pi_{\ell}\bigr\Vert _{L^{\infty}}^{{\frac{1}{2}}} \Vert u\Vert _{L^{4}}^{4}+\Vert u\Vert _{L^{4}} \Vert \pi _{h}\Vert _{W^{-1,\infty}}^{\frac{1}{2}}\Vert \nabla \pi_{h}\Vert _{L^{2}}^{\frac{1}{2}}\bigl\Vert \nabla \vert u\vert ^{2}\bigr\Vert _{L^{2}} \\ &\leq\bigl\Vert \nabla^{2}\pi_{\ell}\bigr\Vert _{L^{\infty}}^{\frac {1}{2}}\bigl(\Vert u\Vert _{L^{4}}^{4}+1 \bigr)+C\Vert \pi_{h}\Vert _{W^{-1,\infty}}^{\frac{1}{2}}\bigl( \Vert u\cdot\nabla u\Vert _{L^{2}}+1\bigr)^{\frac{1}{2}}\Vert u \Vert _{L^{4}}\bigl\Vert \nabla \vert u\vert ^{2}\bigr\Vert _{L^{2}} \\ &\leq\frac{1}{8}\bigl\Vert \nabla \vert u\vert ^{2}\bigr\Vert _{L^{2}}^{2}+\frac{1}{8}\Vert u\cdot\nabla u \Vert _{L^{2}}^{2}+C\bigl(e+\Vert \pi \Vert _{B_{\infty,\infty}^{r}} \bigr)^{\frac {2}{2+r}}\bigl(\Vert u\Vert _{L^{4}}^{4}+1 \bigr)+C. \end{aligned} $$

Inserting (3.9) and (4.4) into (3.8), we obtain (3.13).

By the classical regularity theory of parabolic equations [14], it follows from (1.2), (1.3), (3.13) and (4.4) that

4.5 $$ \begin{aligned}[b] \Vert \nabla n\Vert _{L^{2}(0,T;L^{\tilde{r}})}&\leq C\bigl(1+\Vert un\Vert _{L^{2}(0,T;L^{\tilde{r}})}+\bigl\Vert n \chi(p)\nabla p\bigr\Vert _{L^{2}(0,T;L^{\tilde{r}})}\bigr) \\ &\leq C\bigl(1+\Vert u\Vert _{L^{\infty}(0,T;L^{4})}\Vert n\Vert _{L^{\infty}(0,T;L^{\frac{4\tilde{r}}{4-\tilde{r}}})}+\Vert n\Vert _{L^{\infty}(0,T;L^{\frac{q\tilde{r}}{q-\tilde {r}}})}\Vert \nabla p\Vert _{L^{2}(0,T;L^{q})}\bigr)\hspace{-10pt} \\ &\leq C \end{aligned} $$

for some $3<\tilde{r}<4$ and $\tilde{r}< q$.

Therefore,

4.6 $$ \Vert n\Vert _{L^{2}(0,T;L^{\infty})}\leq C. $$

This completes the proof.
